# A Cell Permeable Peptide Targeting the Intracellular Loop 2 of Endothelin B Receptor Reduces Pulmonary Hypertension in a Hypoxic Rat Model

**DOI:** 10.1371/journal.pone.0081309

**Published:** 2013-11-27

**Authors:** Daniel S. Green, Chamila Rupasinghe, Rod Warburton, Jamie L. Wilson, Christine O. Sallum, Linda Taylor, Achani Yatawara, Dale Mierke, Peter Polgar, Nicholas Hill

**Affiliations:** 1 Department of Biochemistry, Boston University School of Medicine, Boston, Massachusetts, United States of America; 2 Department of Chemistry, Dartmouth College, Hanover, New Hampshire, United States of America; 3 Division of Pulmonary Medicine, Tufts School of Medicine, Boston, Massachusetts, United States of America; Vanderbilt University Medical Center, United States of America

## Abstract

Cell permeable peptides (CPP) aid cellular uptake of targeted cargo across the hydrophobic plasma membrane. CPP-mediated cargo delivery of receptor signaling motifs provides an opportunity to regulate specific receptor initiated signaling cascades. Both endothelin-1 receptors, ETA and ETB, have been targets of antagonist therapies for individuals with pulmonary arterial hypertension (PAH). These therapies have had success but have been accompanied by adverse reactions. Also, unlike the CPP which target specific signaling cascades, the antagonists target the entire function of the receptor. Using the CPP strategy of biased antagonism of the ETB receptor’s intracellular loop 2 (ICB2), we demonstrate blunting of hypoxic pulmonary hypertension (HPH) in the rat, including indices of pulmonary arterial pressure, right ventricular hypertrophy and pulmonary vascular remodeling. Further, ex vivo analysis of the pulmonary artery treated with the IC2B peptide upon injection manifests marked reductions in Akt and ERK activation. Both kinases have been intimately related to cell proliferation and vascular contraction, the hallmarks of PAH. These observations in sum illustrate an involvement of the ETB receptor in HPH and furthermore provide a basis for a novel, CPP-based, strategy in the treatment of PAH, ultimately able to target not only ET-1, but also other factors involved in the development of PAH.

## Introduction

Pulmonary arterial hypertension (PAH) is a disease of multiple etiologies and high morbidity and mortality, characterized by elevated pulmonary artery pressure, cellular proliferation in the pulmonary vasculature with formation of plexiform lesions and right heart hypertrophy and eventually, failure [1]. Concomitant with aberrant cell growth is dysregulation of the G-protein coupled receptors (GPCR) for endothelin-1, ETA and ETB [2]. Individuals with PAH also have increased plasma endothelin-1 (ET-1) levels which further contribute to a contracted state and remodeling of vessels within the lung [3]. The ETA and ETB receptors are therapeutic targets for the dual acting antagonist, bosentan [4]. However, the role of the ETB receptor in the pathogenesis of PAH is not clear. ETB has been shown to mediate vasoconstriction, while certain aspects of ETB action have actually been reported as physiologically beneficial [5-8]. Thus, we sought to determine whether targeted antagonism of ETB signaling in an experimental model has a net inhibitory or contributory effect on the development of pulmonary hypertension.

There are several rodent models of hypoxic pulmonary hypertension (HPH) including monocrotaline as well as chronic hypoxia [9,10]. The hypoxic model has been well-defined with a major component of sustained pulmonary vasoconstriction, combined with structural pulmonary vascular remodeling, leading to chronic elevation of pulmonary arterial pressures and right ventricular hypertrophy [11]. ET-1 plays a role in the pathogenesis of this model of HPH and, in some studies, the ETB receptor has been shown to have ameliorating effects [12]. Which signaling cascade(s) are most important in the induction or continuation of HPH or clinical PAH has not yet been established. Akt and ERK have been reported to participate in animal models of HPH [13]. Akt in isolated smooth muscle cells promotes hypertrophy, proliferation and cell survival [14-16]. ERK has also been shown to promote smooth muscle contraction and proliferation [17]. Therefore, it was important to determine whether our targeted blocking of the IC2 region of the ETB receptor not only influences the development of HPH, but also modifies the activation of these two kinases.

In addition to traditional pharmacological approaches, modulation of the signaling properties of G-protein coupled receptors has also been approached through the use of cell permeable peptides (CPP). CPP are short peptide sequences that can freely cross cell membranes and can facilitate the translocation of various molecular cargos [18]. The ability to transport peptide cargos across the cell membrane provides an opportunity to create peptide motifs which mask, or mimic, functional motifs of GPCRs [19,20]. The effect of CPP is similar to that of biased agonists or antagonists, but does not require interference with ligand binding or receptor internalization. To promote membrane permeability and facilitate internalization, a critical design feature is the use of a SynB3 [21] based-CPP design named SynB3RG. The SynB transporter sequence was derived from protegrin (the SynB peptide vectors), which possess enhanced drug-transport ability across the blood-brain barrier [22]. The mechanism of the SynB cargo delivery system is considered to be an adsorptive-mediated endocytosis process [22]. 

In the present study, we use the hypoxic rat model to determine whether HPH can be attenuated by interfering with ET-1 signal transmission through the second intracellular loop of the ETB receptor. This targeted approach allows us to determine the contribution of the ETB receptor to the development of HPH and whether early Akt and ERK signals are concomitantly altered by the CPP. 

## Materials and Methods

### Animals

Male Sprague-Dawley rats (250-275) were obtained from Taconic Labs (Boston, MA, USA). The Tufts IACUC Committee approved all animal studies. For hypoxic exposures, rats were put in normobaric hypoxia (FIO_2_ 10.5%) or similar normoxic chambers (for controls) for 3 weeks or 24 hr while given intraperitoneal injections of peptide or vehicle injections on day one and weekly thereafter. For a detailed description of the animal procedures see Hypoxia Model and Experimental Design in the Methods S1.

### Synthesis of CPP (IC2B)

For a detailed description of the synthesis of IC2B peptide see Methods S1.

### Determination of Peptide Cell Penetration

5-FAM (5-Carboxyfluorescein) tagged IC2B (IC2B-FAM) peptide (10 uM) in serum-free media was added to pulmonary artery smooth muscle cell cultures grown in 6 well plates. After 30 min at 37°C, to cleave adhering CPP from the cell membranes and to detach the cells from the wells, the cells were washed twice with PBS and then trypsinized and resuspended in PBS as a single cell suspension. The single cell suspension was transferred into tubes at a concentration of 1.5 X 10^6^ cells per ml. Cells were analyzed by flow cytometry on a FacsCalibur (BectonDickinson, Franklin Lakes, NJ). A total of 10,000 gated cells per sample were counted. Data were analyzed using Cytomation Summit software (Cytomation Inc., Fort Collins, USA). As controls, cells were incubated with peptide-free medium. 

### Determination of IC2B peptide Cell Penetration in the isolated Pulmonary Artery

Pulmonary arteries were isolated from adult rats (250-275 g) washed in sterile HBSS twice. The vessels were then cut open so as to allow maximum exposure of the internal and external vessel to the peptide. The vessels were then exposed to 5-FAM tagged IC2B peptide (10 uM) in HBSS without phenol red for 1 hr at 37°C in the dark. Control vessels were placed in the same media without the 5-FAM tagged IC2B peptide. Following exposure the vessels were washed in fresh HBSS twice and placed in 4% NBF overnight then paraffin embedded and 5 um sections made. Sections were viewed on Zeiss (Thornwood, NY) fluorescence microscope and images recorded. 

### Determination of IC2B peptide Cell Penetration in vivo

5-FAM tagged IC2B peptide (10 uM) was administered IP, following 2 hours the animal was sacrificed and the lungs inflated with OCT and then frozen. Cryosections were made, observed and recorded on Zeiss fluorescence microscope

### Histological Analysis of Pulmonary Artery

Morphological analysis was done following lung fixation and embedding. Transverse sections, 5 um were cut from left lung midzones of paraffin-mounted lungs. Sections were stained with van Gieson elastic stain. Vessels below 125 um diameter were examined. Vessels were graded as none (<25%), partial (25%-75%), and fully (>75%) muscularized. Fifty vessels per slide were graded by readers blinded to the experimental group. 

### Ex Vivo Pulmonary Artery Kinase Signaling

The pulmonary artery was isolated from treated and control animals, cut into 2 mm rings and placed in cold serum-free DMEM on ice. By carefuly clearing the vessel slices (rings) of any remaining adventitia or intima we insured that the rings consisted principally of smooth muscle. Three to four rings were placed in 24 well plates with pre-warmed serum-free DMEM and incubated as described in the figure legends. Following incubation the rings were washed with cold PBS, placed in cryogenic vials, and immediately snap frozen in liquid nitrogen. The frozen vials were stored at –80°C. For western blotting, the rings were thawed on ice in 100 ul of RIPA buffer with protease and phosphatase inhibitors included to maintain the generated phosphorylated proteins. The rings were then dissociated on ice using 17 gauge needles. After dissociation, the samples were centrifuged at 10,000 RPM for 30 min at 4°C and the supernatant was used for western blotting procedures. 

### Western Blotting

Protein concentrations were determined by BCA method. SDS-PAGE was run with 4% stacking gels and 10% separating gels. SDS-PAGE separations were transferred on nitrocellulose membranes (BioRad, Hercules, CA) at 100 V and 4°C for 1 h. Membranes were washed 3 times for about 5 min with TBS-T (20 mM Tris, 150 mM NaCl, 0.1% Tween 20, pH 7.6), blocked at room T for 1 h with 5% powdered milk in TBS-T, washed again 3 times, for 5 min, and incubated in 1° rabbit antibody at 4°C overnight in 1:1000 phospho-p44/p42 MAPK Thr202/Tyr204 (pERK) (#9101), 1:2000 phospho-Akt Ser473 (pAkt) (#4060), 1:1000 total Akt (tAkt) (#9272) or 1:1000 total ERK (tERK) (#9102) dilutions plus 5% BSA in TBS-T. All 1° antibodies were purchased from Cell Signaling Technologies (Cell Signaling, Danvers, MA). Blots were washed again in TBS-T followed by 1 h incubation in the corresponding anti-rabbit 2° Ab (Santa Cruz Biotechnology, Santa Cruz, CA) diluted 1:10,000 TBS-T. Blots were then washed 3 times in TTBS-T, and developed in detection reagents from Thermo Scientific (Rockford, IL) for 1 min before exposure to autoradiography film. Densitometry on band intensities was performed using the NIH ImageJ image analysis software. 

### Statistics

Mean values were calculated for each group of rats. Differences in mean values between normoxic and hypoxic animals were compared by two-way ANOVA. Where significant differences were observed, pairwise comparisons were done using the Student–Newman-Keuls method. Differences in percent muscularization of pulmonary vessels were analyzed by proportion comparison and Yates correction. Data are expressed as means + SEM. Differences were considered significant at P<0.05. Statistical analysis was done using SigmaStat (Systat, Point Richmond CA).

## Results

### Cellular and tissue penetration of the CPP Molecule

**Figure 1:**
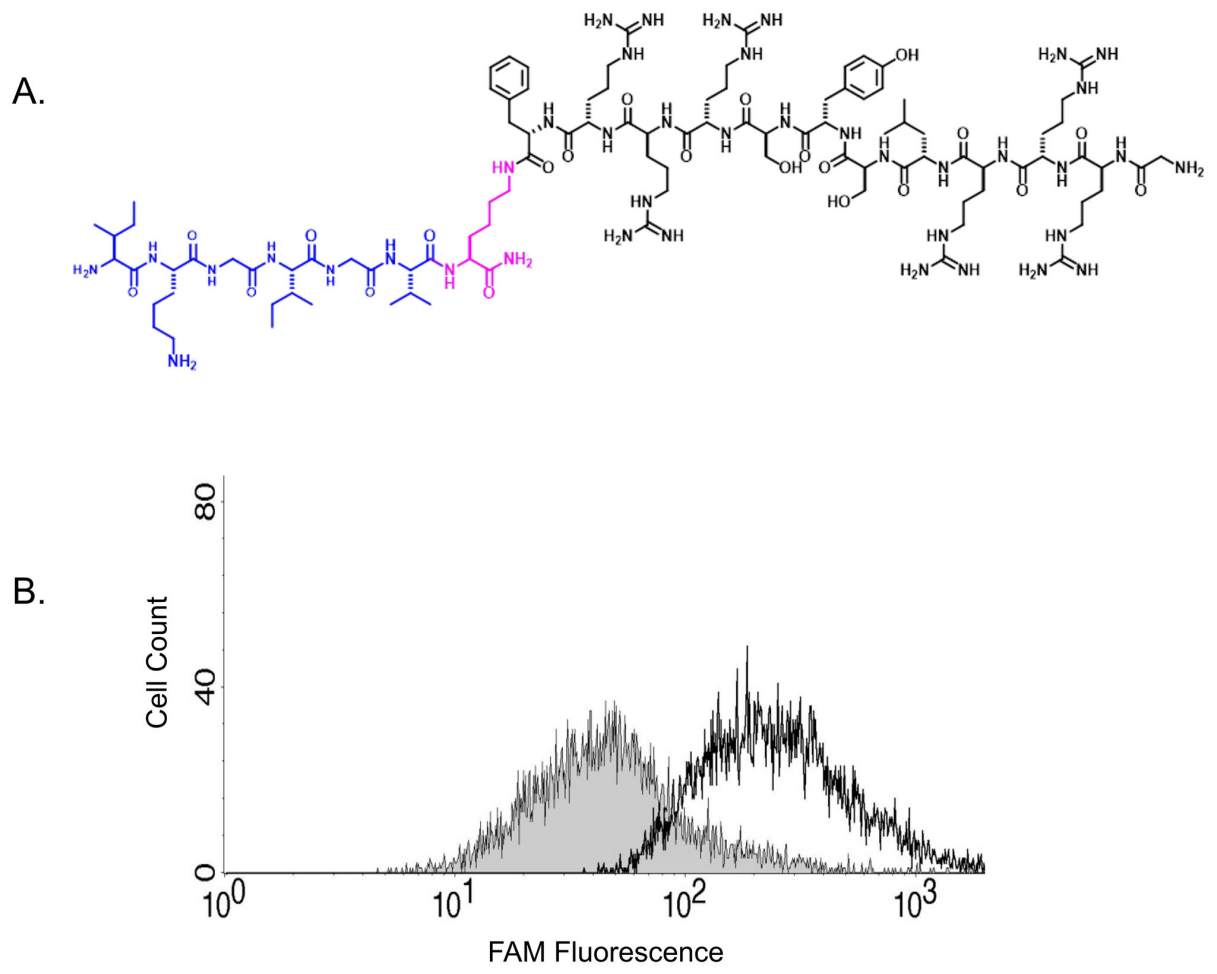
Uptake of 5-FAM tagged IC2B peptide into pulmonary artery smooth muscle cells. The chemical structure of SynB3RG transporter sequence attached to IC2 loop of the ETB receptor is illustrated in (A). Flow cytometry shows that 5-FAM tagged IC2B peptide entered the cells efficiently after 20 mins of exposure (B).

**Figure 2:**
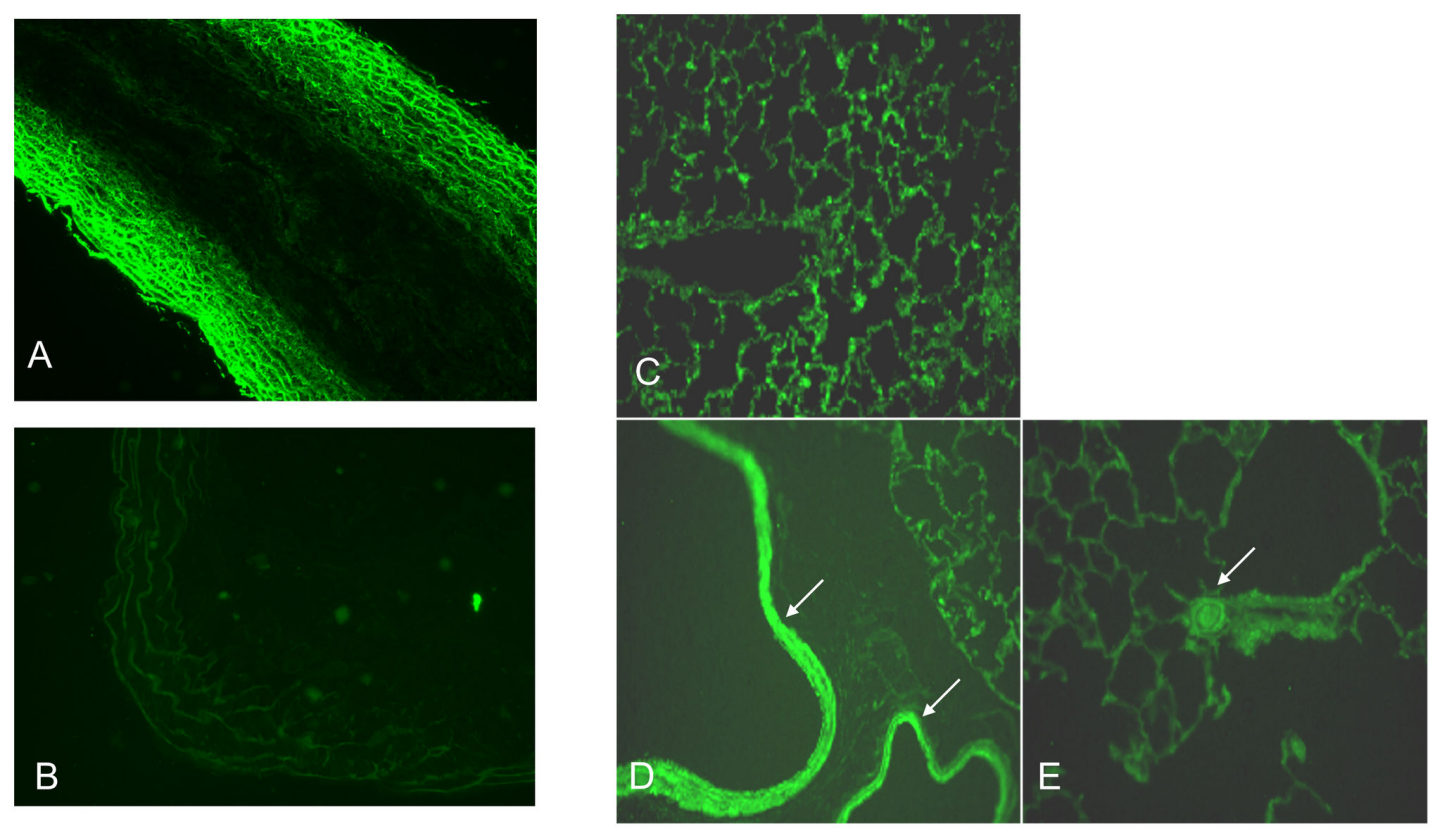
Penetrance of 5-FAM tagged IC2B peptide into rat pulmonary artery tissue. *Ex* *vivo* vessel tissues were imaged with a fluorescence microscope at 40X magnification after treating with medium containing 5-FAM tagged IC2B peptide (A) or sham (B) for 1 hr. *In* *vivo* penetrance was determined by taking images of normal rat lung with no injected IC2B-FAM (C), a lung from a rat IP injected with the IC2B 5-FAM (D,E). Arrows show staining of SMC in airways and large vessels (D) and small arterioles (E).

A CPP mimetic of the IC2 loop of the ETB receptor is drawn schematically in Figure **1A**. As illustrated, the IC2 loop of the ETB receptor was attached to the SynB3RG transporter sequence using a lysine side-chain introduced at its C-terminus (Figure **1A**) and termed IC2B. To test the ability of the IC2B to penetrate cells a FAM labeled-derivative was synthesized termed IC2B-FAM. The FAM label was introduced at the side chain of the lysine residue of the SynB3R component of the ligand (Figure **1A**). Pulmonary artery smooth muscle cells incubated with the IC2B-FAM were analyzed for fluorescence using flow cytometry. Results are illustrated in Figure **1B**. The IC2B-FAM was efficiently inserted into cells within twenty minutes. Tissue penetration of the IC2B was also tested in intact rat pulmonary arteries. Pulmonary arteries after excision were incubated with IC2B-FAM at 10 mM for 20 minutes. Strong fluorescence is seen in the presence of IC2B-FAM whereas little to no fluorescence, indicative of autofluorescence, is seen in control vessels (Figure **2A,B**). The layers of fluorescence in the treated vessels appear to be smooth muscle. Following an IP injection, the IC2B, attached to FAM also penetrates the lung vasculature and is highly selective for smooth muscle cells including the large and small pulmonary arteries as illustrated in Figure **2C,D,E**.

### Hypoxia and the physiologic effect of the IC2B CPP

**Figure 3:**
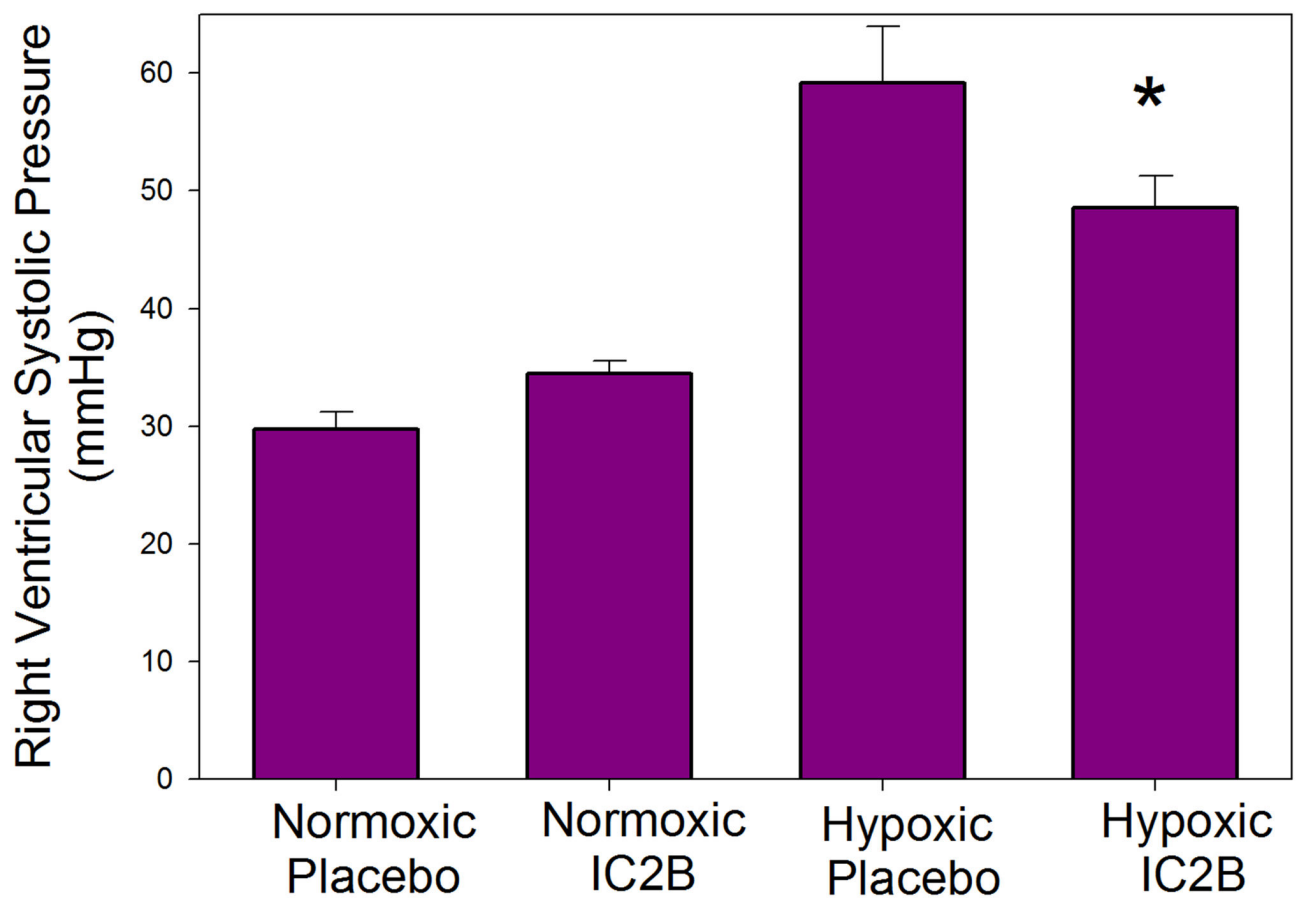
Effect of the IC2B peptide on right ventricular systolic pressure (RSVP) in hypoxic rats. Rats were treated with hypoxia or room air for 3 weeks while given injections of peptide or saline on day one and weekly thereafter. Data are means + SEM N=6-10 per group . * P<0.05 vs hypoxic saline.

**Figure 4:**
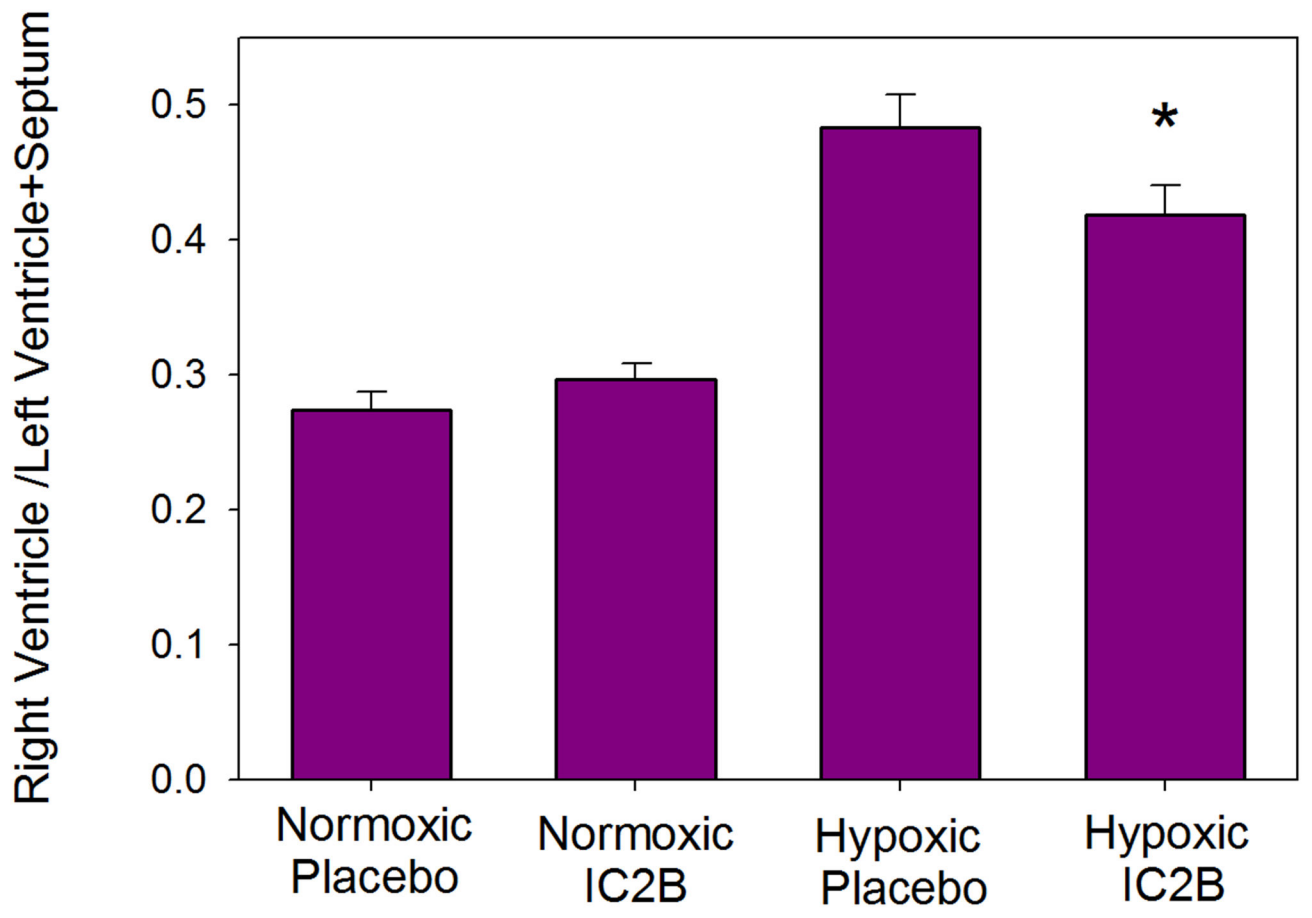
Effect of the IC2B peptide on hypertrophy in hypoxic rats. Rats were treated with hypoxia or room air for 3 weeks while given injections of peptide or saline on day one and weekly thereafter. Hypertrophy was calculated by the right ventricular / left ventricle + septum (RV/LV+S) ratios. Data are means + SEM N=6-10 per group * P<0.05 vs hypoxic saline.

The rat hypoxia model of PAH was used as described in the Methods section. After 3 weeks of exposure hypoxic rats treated with the IC2B peptide showed a significant decrease in the right ventricular systolic pressure (RVSP) vs. saline treated hypoxic controls (48.6+2.7 vs 59.1+4.8 p=0.029) (Figure **3**). Right ventricular pressures were similar in both the IC2B peptide and control normoxic animals, indicating no effect of the peptide in normoxic animals. Furthermore, as shown in Figure **4**, there was reduced hypertrophy in the IC2B peptide treated hypoxic right ventricle as measured by RV/LV+S compared with RVs from saline-treated hypoxic controls (0.398+.021 vs 0.483+.025 p=.005). Systolic and diastolic systemic blood pressure was unaffected by treatment with the IC2B or hypoxia; nor was there any statistically significant difference in cardiac index or LV+S/BW among the groups (Table **1**). Although not statistically significant and not associated with any alteration in RVSP, there was a slightly lower cardiac index in the normoxic rats treated with the IC2B.

**Table 1:** Body weight (BW), systemic blood pressure (systolic and diastolic), and left ventricle + septum/ body weight (LV++S/BW) of rats exposed to 3 weeks hypoxia or room air and injected with IC2B peptide or saline.

	BW (g)	Systolic (mm Hg)	Diastolic (mm Hg)	Cardiac Index (ml/min/kg)	LV+S/BW
Normoxic Saline	350 + 16	135 + 4	105 + 5	190 + 12	2.17 + 0.04
Normoxic IC2B	373 + 14	134 + 5	100 + 6	142 + 4	2.17 + 0.09
Hypoxic Saline	311 + 5*	142 + 4	115 + 3	205 + 39	2.04 + 0.05
Hypoxic IC2B	315 + 4*	155 + 4	123 + 3	198 + 19	2.14 + 0.09

Data are means + SEM N=6-10 per group * P<0.05 vs normoxic

### Histological analysis of small pulmonary vessels

**Figure 5:**
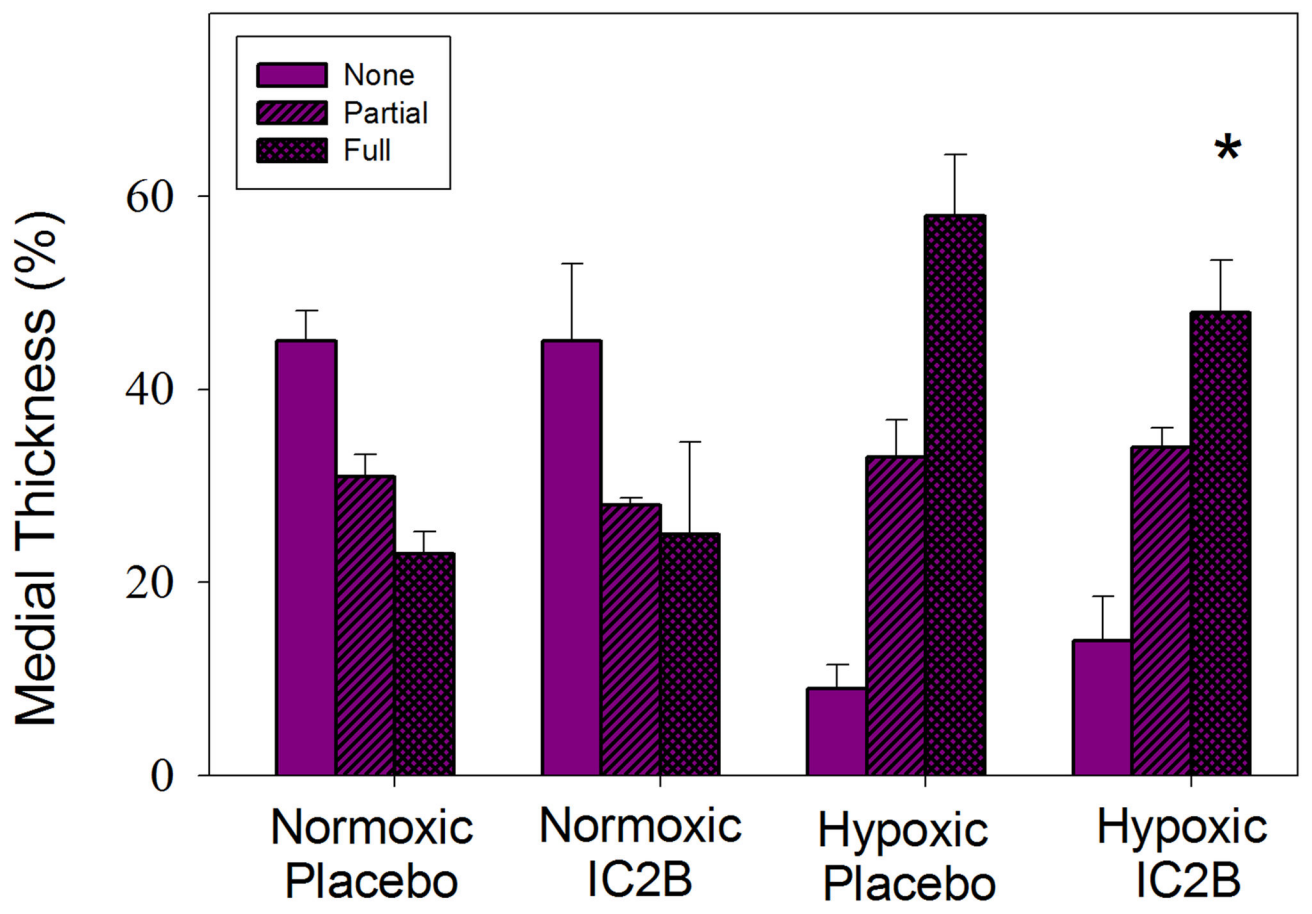
Effect of the IC2B peptide on vessel muscularization. Percentage of peripheral pulmonary vessels that were fully, partial, or nonmuscularized (none) of rats exposed to 3 weeks hypoxia or room air and injected with IC2B peptide or placebo. Data are means + SEM N=6-10 per group * P<0.05 vs hypoxic saline full.

Normoxic saline-treated and IC2B-treated animals displayed no significant differences in percent muscularization of small pulmonary vessels (Figure **5**). The hypoxic animals treated with saline showed the typical response of a significant increase in fully muscularized vessels versus the normoxic groups. Between the hypoxic groups, the IC2B-treated vessels had significantly blunted increases in per cent fully muscularized vessels compared to saline controls. 

### Toxicology of IC2B

Body weights of both hypoxic groups were significantly less than normoxic rats, but there was no significant difference between the IC2B groups and their respective controls, nor was there evidence of extremity edema or ascites, suggesting that IC2B did not induce fluid retention. Analysis of standard liver function studies as well as blood urea nitrogen showed no evidence of hepatorenal toxicity attributable to IC2B. Aspartate aminotransferase increased with exposure to hypoxia compared to normoxic rats, but no further change occurred with 3 weeks’ administration of the IC2B peptide (Table **2**). 

**Table 2:** Blood indices to assess for hepatorenal toxicity of IC2B.

	Normoxic	Hypoxic	Hypoxic + CPP
Alkaline Phosphatase	179 + 20	195 + 31	176 + 16
ALT	25 + 4	30 + 10	33 + 6
AST	48 + 3	73 + 8 *	81 + 8 *
Creatine Kinase	163 + 46	236 + 90	225 + 38
GGT	2.0 + 0.0	3.0 + 0.0	2.4 + 0.6
Albumin	2.9 + 0.1	2.9 + 0.2	2.8 + 0.1
Total Protein	5.6 + 0.2	5.2 + 0.2	5.0 + 0.1
Total Bilirubin	0.1 + 0.0	0.1 + 0.0	0.1 + 0.0
Blood Urea Nitrogen	18.8 + 2.8	26.3 + 8.4	20.0 + 2.2

Abbreviations: ALT = alanine aminotransferase; AST = aspartate aminotransaminase; GGT = gamma glutamyl transpeptidase. *p < 0.05 vs normoxic.

### Effect of IC2B on ETB receptor promoted kinase activation during hypoxia

**Figure 6:**
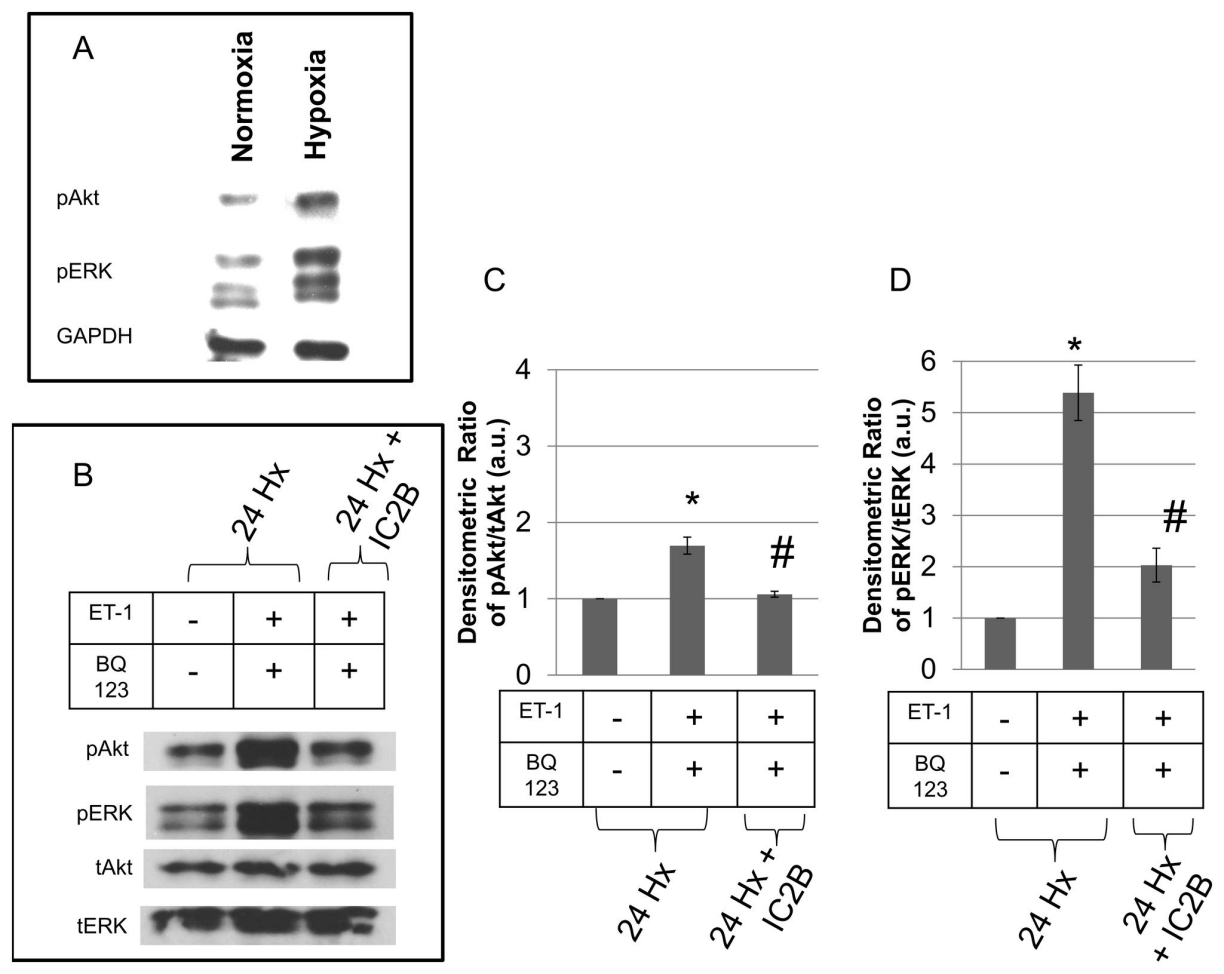
Effect of the IC2B peptide on pulmonary artery Akt and ERK signaling. Rats were treated with hypoxia and injected with IC2B peptide for 24 h. Pulmonary artery rings were isolated and pre-incubated with or without 1uM BQ123 for 30 mins. Then, the rings were treated with or without 10 nM ET-1 for 20 min. Protein lysates were probed for pAkt, pERK, tAkt and tERK. Western blot analysis comparing basal pAkt and pERK levels in pulmonary artery rings from normoxic and hypoxic rats (A). Western blot analysis of rat pulmonary artery rings from hypoxic rats injected with the IC2B peptide (B). Bar graphs illustrate the densitometric ratios of pAkt (C) and pERK (D) normalized to tAkt and tERK, respectively. Blots are representative of triplicate experiments. Data are means + SEM N=3 * P<0.05 vs hypoxic control. # P<0.05 vs hypoxic ET-1 plus BQ123. a.u=arbitrary unit.

Using the same protocol for the hypoxic experiments as above, rats were subjected to hypoxia for 24h in the presence or absence of injected IC2B peptide. After the 24h exposure, pulmonary arteries were excised and ERK and Akt activation was determined in tissue slices. Hypoxia increased the levels of both phosphorylated ERK and Akt in pulmonary artery slices from hypoxic compared to normoxic rats (Figure 6A). Other slices were treated with BQ123, an antagonist of ET-1 binding to the ETA receptor, as a means of focusing on the action of the ETB receptor. This inhibitor was formerly shown to block over 90% of ETA promoted ERK activation while not affecting ETB promoted signaling [2]. Under these conditions, as illustrated in Figure **6B,C,D**, ET-1 markedly activated Akt and ERK in pulmonary arteries (as determined by the ratio of phosphorylated to total protein) from rats subjected to hypoxia. This activation was markedly blunted by the IC2B peptide. 

## Discussion

The principal aim of this study was to examine the possibility that a CPP blocking a narrower signaling segment of a receptor than that of a receptor antagonist could be used as a means to study the mechanisms of PAH as well as a potential therapeutic target for this disease. Within this aim we elected to focus on the actions of the ETB receptor in a rat model of pulmonary hypertension. Prior work has identified the ETA receptor as the principal ET-1 contributor to the pathology of PAH, while the role of ETB has been far less understood. Currently, available endothelin-1 receptor antagonists are dual receptor blockers. These have provided therapeutic benefits. However this treatment has come at a cost because the antagonists, with their wide-ranging blocking effects, have adverse side effects including liver toxicity and fluid retention [23]. Based on these toxic effects, targeted modulation of ETB receptor signaling, rather than its entire blockade, would provide a novel and interesting approach toward the treatment of pulmonary hypertension. It would also provide insight into the role of ETB in HPH pathogenesis. In previous studies we showed that CPP-mimicking motifs within the intracellular faces of GPCR can alter signal cascades by such vasoactive effectors as angiotensin and endothelin-1 [19,20].. Here, we synthesized a CPP containing a copy of the intracellular second loop of the ETB receptor. 

Deciphering the contribution of the ETB receptor to the pathogenesis of pulmonary hypertension has been complicated by the fact that both vasodilatory and vasoconstrictor actions have been ascribed to it [24]. Prior studies on the role of the ETB receptor in rodent pulmonary hypertension have demonstrated an exacerbation of pulmonary hypertension in the spotting-lethal rat, which carries a deletion of ETB, in both the hypoxic [25] and monocrotaline [26] PAH models. More recently, similar findings have been reported in mice genetically deficient in the ETB receptor when exposed to hypoxia [27]. Our approach to receptor blockade is very different than the genetic deletion approach used in the earlier rodent studies. Targeting of limited signal cascades confers specificity of blockade and provides an opportunity to target deleterious actions while leaving desirable receptor effects intact, such as the ET-1 clearance function of ETB. Also, our immunofluorescent studies on pulmonary arterial distribution of the IC2B in both ex vivo and in vivo preparations suggest that it penetrates cells in the vascular media where the ETB may have important vasoconstrictive/proliferative effects. 

Using the hypoxic rat model of PAH, we showed that the presence of the IC2B ameliorated the development of HPH. We found that this peptide hindered ET-1 promoted ERK and Akt phosphorylation in rat pulmonary artery smooth muscle. These two kinases are important participants in vascular hypertrophy and both are upregulated in HPH [28]. We also demonstrated in the hypoxic rat model of PAH that the peptide mitigates PAH manifestations. Specifically, the IC2B significantly decreased right ventricular systolic pressure and right ventricular hypertrophy. Histologic examination of the small pulmonary vessels of hypoxic animals showed that the IC2B also significantly decreased fully muscularized vessels compared to controls. The protective effects were pulmonary selective since there was no reduction in systemic pressures. These findings illustrate that, in this model, the ETB receptor does indeed contribute to the pathogenesis of HPH. Additionally, we demonstrate that CPPs may have therapeutic potential for pulmonary hypertension. 

Our results are surprising considering other previous studies which indicated that a blockade of the ETB receptor exacerbates pulmonary hypertension in rodent models (25-27). This suggests that blockade of specific signaling components of the ETB receptor may have different physiologic effects. For example, it has long been known that the ETB receptor in endothelial cells promotes release of vasodilators such as nitric oxide [29] and clears circulating ET1 [30]. The IC2B could leave these functions intact while blocking the vasoconstrictor actions mediated by the ETB receptor on smooth muscle cells, thus ameliorating hypoxic pulmonary hypertension, as we observed. 

Our study has some limitations. While the IC2B clearly mitigates HPH manifestations, the dosage and frequency of injection may not have been optimal. Future studies on pharmacokinetics of the CPPs will be necessary to optimize these factors. Also, although the imunohistochemistry studies we performed demonstrate staining of vascular media, our methods did not permit differentiation of IC2B actions on smooth muscle cells vs endothelial cells. Further, our signaling studies are based on observations from more proximal pulmonary arteries and may vary from the distal resistance vessels which we were unable to dissect from intact lungs due to technical limitations. In the same context, while we focused our signaling determinations on tissue rings containing the media by meticulously scraping off the adventitia and intima a very small fraction of endothelial and fibroblastic cells may have remained in the rings. 

In conclusion, our findings indicate that CPPs are capable of specific physiologic blockade, such as the vasoconstrictor actions of the ETB receptor. Thus, unlike treatment with standard pharmacologic receptor antagonists which block the entire receptor action regardless of beneficial or deleterious actions, the CPP approach can be much more specifically targeted at deleterious actions and could well avoid off-target organ toxicity. However, ET-1 and its ETB receptor are only components of a number of other molecular contributors to the etiology of PAH. Clearly, realizing the therapeutic potential of CPPs will require considerably more work and possibly targeting of multiple pathways simultaneously in future studies of these molecules.

## Supporting Information

Methods S1 (DOCX)
