# Supplementary material for: A Cell Permeable Peptide Targeting the Intracellular Loop 2 of Endothelin B Receptor Reduces Pulmonary Hypertension in a Hypoxic Rat Model
Source: PLoS One. 2013 Nov 27;8(11):e81309. doi: 10.1371/journal.pone.0081309 (PMC3842336; doi:10.1371/journal.pone.0081309)
Supplement: Methods S1 — (DOCX) [file pone.0081309.s001.docx]

**Supporting Information**

**Materials and Methods**

*Hypoxia Model* and *Experimental Design*

Male Sprague-Dawley rats (250-275) were obtained from Taconic Labs (Boston, MA, USA). The Tufts Boston IACUC Committee approved all animal studies. Animals were exposed to hypobaric hypoxia (0.5 atm) for 3 week or 24 hr. Cages were changed twice weekly with food and water given Ad Libitum. Interperitoneal injections of peptide were given one d before start of hypoxia then given once per week for duration of exposure for a total of four injections. Dose of IC2B (2 mg) was given in 300 ul of sterile saline. Following three weeks of hypoxia, animals were removed to room air and anesthetized with ketamine/pentobarbital (60 mg/kg IM 20 mg/kg IP) and body weights (BW) recorded. The right ventricular systolic pressure was measured by inserting a liquid filled catheter connected to a pressure transducer (Grass P23) into the right external jugular vein and advanced into the right ventricle. Pressure wave forms are recorded (PowerLab ADInstruments, Colorado Springs, CO) for 5 min and an average systolic pressure recorded. The right carotid artery was isolated and a catheter inserted and a systemic pressure recorded for 5 min and systolic and diastolic averages recorded. Cardiac output was determined using the thermodilution technique. A thermal probe and polypropylene catheter were placed in the aortic arch and right ventricle, respectively. Room temperature saline (200 µl) injections were made into the right ventricle and thermodilution curves were recorded after each injection using a PowerLab computer (AD Instruments, CO). Cardiac output was calculated as a function of the area under the curve, averaged in triplicate.

Following pressure and output measurements the abdomen is opened and blood collected by the inferior vena cava. The animals are then euthanized by exsanguination. The chest is then opened and the pulmonary artery and trachea are catheterized and heart and lungs are removed en bloc. The right lung is ligated and removed and frozen in liquid nitrogen. The left lung is fixed (4% formalin) at 23 cm H_2_O tracheal and 75 cm H_2_O for the pulmonary artery. Following 30 min fixation, the lung is removed and placed in formalin for later embedding. The heart is removed and the right ventricle (RV) is separated from the left ventricle and septum (LV+S). This was done in several animals per group. The remaining animals had heart and lungs removed to separate and weigh RV and LV+S before freezing. RV/LV+S ratios are calculated by dividing RV weight by LV+S weight, RV/BW and LV+S weights are also calculated.

*Synthesis of IC2B (CR3110)*

The intracellular second loop of the ETB receptor was attached to a cell permeable sequence to produce the IC2B (Ile-Lys-Gly-Ile-Gly-Val-Lys[SynB3-Arg-Gly]-CONH2). All the compounds were synthesized by Fmoc-based solid-phase peptide synthesis (SPPS) protocols employing microwave heating (CEM Discover S-class microwave synthesizer), with modifications as required to generate the modified analogs. Compound IC2B was synthesized on Rink-Amide-ChemMatrix resin (*N* mmol, 0.6 mmol/g, P/N no. 7-600-1310-25). First coupling of Dde-Lys(Fmoc)-OH was carried out using 5 equiv Dde-Lys(Fmoc)-OH, 5 equiv HBTU in DMF, MW, 70 °C, 6.5 min followed by Fmoc deprotection using piperidine (25% v/v)-HOBt(1.25% w/v)-DMF, MW, 70 °C, 6.5 min. Then the SynB3R cell transported sequence was synthesized. This was carried out by sequential coupling of Fmoc-Arg(Pbf)-OH x 3, Fmoc-Leu-OH, Fmoc-Ser(tBu)-OH, Fmoc-Tyr(tBu)-OH, Fmoc-Ser(tBu)-OH, Fmoc-Arg(Pbf)-OH x 3, and Fmoc-Phe-OH using 5 equiv Fmoc-aa-OH, 5 equiv HBTU in DMF, MW, 70 °C, 6.5 min for amino acid coupling and piperidine (25% v/v)-HOBt(1.25% w/v)-DMF, MW, 70 °C, 4.5 min, for Fmoc deprotection. This was followed by coupling of Boc-Gly-OH using 5 equiv Boc-Gly-OH, 5 equiv HBTU in DMF, MW, 70 °C, 6.5 min. After the coupling of Boc-Gly-OH group, deprotection of *N*-terminal Dde group of lysine side-chain was carried out using 5% hydrazine monohydrate in DMF, 30 min at RT. Following Dde group deprotection the IC2 loop of the ETB receptor was assembled. This was carried out by sequential coupling of Fmoc-Ile-OH, Fmoc-Lys(Boc)-OH, Fmoc-Gly-OH, Fmoc-Ile-OH, Fmoc-Gly-OH, and Fmoc-Val-OH were using 5 equiv Fmoc-aa-OH, 5 equiv HBTU in DMF, MW, 70 °C, 6.5 min for amino acid coupling and piperidine (25% v/v)-HOBt(1.25% w/v)-DMF, MW, 70 °C, 4.5 min, for Fmoc deprotection. The final side chain deprotection and resin cleavage was carried out by adding the cleavage solution (5x resin volume, TFA/TIS (triisopropylsilane)/thioanisole/anisole (94:2:2:2, v/v)) and reacting the resin using MW, at 40 °C for 45 mins. The cleavage solution was later collected and separated into two 10 mL test tubes, followed by addition of ethyl ether (-20 °C). After thoroughly mixing the contents, peptide precipitate was centrifuged (8 min, 6000 rpm), and the supernatant was decanted. At last, the peptide was dissolved in distilled water (5-10 mL), frozen, and lyophilized until a white powder was obtained. Compound IC2 peptide was purified by RP-HPLC, and molecular mass confirmed by MALDI-TOF mass spectroscopy. MALDI-TOF-MS (m/z): C_101_H_178_N_40_O_22_; calcd, 2304.7 [M]; found, 2305.7 [M+H]^+^.
